# 3D Multicellular Tumor Spheroids in a Microfluidic Droplet System for Investigation of Drug Resistance

**DOI:** 10.3390/polym14183752

**Published:** 2022-09-08

**Authors:** Sang Ik Lee, Yoon Young Choi, Seong Goo Kang, Tae Hyeon Kim, Ji Wook Choi, Young Jae Kim, Tae-Hyung Kim, Taewook Kang, Bong Geun Chung

**Affiliations:** 1 Department of Mechanical Engineering, Sogang University, Seoul 04107, Korea; 2 Institute of Integrated Biotechnology, Sogang University, Seoul 04107, Korea; 3 Department of Biomedical Engineering, Sogang University, Seoul 04107, Korea; 4 School of Integrative Engineering, Chung-Ang University, Seoul 06974, Korea; 5 Department of Chemical and Biomolecular Engineering, Sogang University, Seoul 04107, Korea

**Keywords:** microdroplet, concentration gradient generator, multicellular cancer spheroid, anti-cancer drug screening

## Abstract

A three-dimensional (3D) tumor spheroid model plays a critical role in mimicking tumor microenvironments in vivo. However, the conventional culture methods lack the ability to manipulate the 3D tumor spheroids in a homogeneous manner. To address this limitation, we developed a microfluidic-based droplet system for drug screening applications. We used a tree-shaped gradient generator to control the cell density and encapsulate the cells within uniform-sized droplets to generate a 3D gradient-sized tumor spheroid. Using this microfluidic-based droplet system, we demonstrated the high-throughput generation of uniform 3D tumor spheroids containing various cellular ratios for the analysis of the anti-cancer drug cytotoxicity. Consequently, this microfluidic-based gradient droplet generator could be a potentially powerful tool for anti-cancer drug screening applications.

## 1. Introduction

Typically, cancer researchers depend on two-dimensional (2D) in vitro or animal studies to examine the complex mechanisms of cancer cell behavior. In vitro cell culture systems are essential tools for biological research. In conventional 2-dimensional (2D) monolayer culture systems, the cellular activities are transformed and the loss of their specific functions can occur due to a lack of interactions between the cellular and extracellular microenvironments [1]. Despite their limitations, 2D monolayer cell cultures are still used for most cell cultures. To better mimic the physiological tissue and further ameliorate the predicting capability, three-dimensional (3D) cell culture systems have obtained increasing attention for building up the in vitro model, especially in cancer research [2,3]. Recently, a multicellular tumor spheroids (MCTs) model has gained increased recognition as an intermediated phase between in vitro 2D cultures and in vivo studies in cancer research [4,5]. MCTs closely mimic the in vivo tumor’s specific features, such as the organization of tumor structure, the gradient oxygen, pH, and nutrients [6,7]. Moreover, MCTs exhibited similarity to in vivo tumors in growth kinetics, metabolic rates, and resistance to chemotherapy and radiotherapy as compared to 2D monolayer cell cultures [8,9]. Furthermore, the MCTs model offers the opportunity to investigate cell–cell and cell–matrix interactions by co-culturing the histologically relevant cells that constitute the tumor tissue. As a result, MCTs can offer enhanced biological relevance to in vivo tumors and also contribute to a better understanding of tumor biology.

Some strategies to generate MCTs include hanging drop [10], a spinner flask or rotary cell vessel [11], a non-adherent surface [12], a scaffold, or extracellular matrix (ECM) gel structures [13]. However, although numerous methods have been used for the preparation of MCTs, there are still some limitations, such as low generation yield, the irregular size of spheroids, inaccurate experimental results, and time consumption. To overcome these limitations, a number of researchers have focused on the development of microfluidic-based techniques, enabling the control of a small sample volume, cost-effective fabrication, and high-throughput screening (HTS) [14,15]. A number of microfluidic systems have been applied to multicellular spheroids culture and drug screening over prolonged periods including microwells, microbubbles, and droplets [16,17,18]. In particular, flow-focusing microfluidics has recently obtained a significant interest in biomedical engineering fields, as they can generate liquid droplets with size-controlled, monodispersed size, and shape with high yields [19,20]. Additionally, the microdroplet system can precisely control the number of cells and cellular contents encapsulated in each droplet [21].

In this study, we report the development of a high-throughput tree-branched microfluidic droplet system for individual multicellular spheroids formation to investigate the relationship between cell–ECM interaction and anticancer drug resistance. This tree-branched microfluidic droplet system allows the generation of spheroids with different cellular percentages that are easy to gather and allows the analysis of the multicellular spheroids sequentially. We encapsulated two different types of cells: MCF-7 breast cancer and NIH 3T3 fibroblast cells. The multicellular spheroids were transferred right after formation and were maintained for up to 5 days on the culture dish with a minimal adverse effect on cell viability. Next, the multicellular spheroids were exposed to various concentrations of anticancer drugs, followed by cytotoxicity analysis, to determine the efficiency of anticancer drug screening. Moreover, we mimicked the in vivo solid tumor structure with heterogeneous cell types by encapsulating two different types of cells to investigate the relevance between the ECM contents and drug resistance.

## 2. Materials and Methods

### 2.1. Fabrication of the Microfluidic Gradient Droplet Device

The microfluidic gradient droplet device was fabricated using a standard soft lithography process as previously reported [22,23]. Briefly, a photomask of the device was designed by 2D CAD software (AutoCAD, Autodesk Inc., San Rafael, CA, USA), and the oxidized silicon wafer was spin-coated with a SU-8 photoresist (Microchem Corp, Westborough, MA, USA) at 2000 rpm for 60 s to form a thickness of 50 μm. After pre-exposure baking at 95 °C for 20 min, the wafer was exposed to ultraviolet (UV) light for 10 s through the photomask. The resulting wafer was developed using a SU-8 developer (Microchem Corp, Westborough, MA, USA) and cleaned with deionized (DI) water and nitrogen gas. Polydimethylsiloxane (PDMS, Dow Corning, Midland, MI, USA) was mixed in a ratio of 10:1 (monomer: catalyst), degassed, poured onto the wafer, and then cured at 80 °C for 2 h. The PDMS mold was peeled off and bonded onto a glass slide (Marienfeld, Cologne, Germany) using oxygen plasma treatment (Femto Scientific, Hwaseong, Korea). The fabricated device was treated with chlorotrimethylsilane (Sigma-Aldrich, St. Louis, MO, USA) and stored at room temperature before use.

### 2.2. Computational Simulation

The numerical simulation was conducted via the computational fluid dynamics (CFD) module and particle tracing module in COMSOL Multiphysics 6.0. Particles as cell models were modeled by conferring different diameters of 7 and 12 μm, respectively. The particle-tracing process was simulated with a 2D model, where the cross-section of the actual components and geometries were reflected. A CFD module was employed to compute the flow distribution in a steady-state, which was determined by calculating incompressible Navier–Stokes equations as follows [24]:

(1) $$\rho \left(\frac{\partial u}{\partial t}+u\cdot \nabla u\right)=-\nabla p+\mu \nabla^{2}u$$

where *u* refers to the velocity vector, *ρ* and *μ* are the density and viscosity of the fluid, *p* is pressure, and *g* is the gravitational acceleration vector. The particles were released from each inlet with the same linear velocity (1 m/min for 10 μL/min) and forced to migrate along with the fluid flow. A time-dependent study was computed to illustrate the migration of particles in terms of time via a particle tracing module, conferring the result from the CFD module. Assuming that the flow in the microfluidic device is a creeping flow, the governing equation for tracing particles can be described below [25,26]:

(2) $$\frac{d\left(m_{p}v\right)}{dt}=\frac{1}{\tau_{p}}m_{p}\left(u-v\right)$$

(3) $$\tau_{p}=\frac{\rho_{p}d_{p}{}^{2}}{18\mu }$$

where $m_{p}\;$is the particle mass, v and u are the velocity vectors of particles and fluid, respectively, $\tau_{p}\;$is the particle velocity response time, $\rho_{p}\;$is the particle density, and $d_{p}\;$is the particle diameter. Droplet generation was excluded in this simulation to reduce the calculation complexity and was substituted by inputting back pressure at the outlets. Based on a previous reference, we set the back pressure to a magnitude of 100 Pa and a frequency of approximately 1–2 Hz.

### 2.3. Fluorescent Cell Lines for 3D Tumor Spheroids

Breast cancer cell line (MCF-7) cells and mouse fibroblast (NIH-3T3) cells from Korean Cell Line Bank were cultured in Dulbecco’s Modified Eagle Medium (DMEM, Thermo Fisher Scientific, Burlington, MA, USA) with 10% fetal bovine serum (FBS, Thermo Fisher Scientific, Burlington, MA, USA) and 1% penicillin-streptomycin (Thermo Fisher Scientific, Burlington, MA, USA) in a humidified incubator with 5% CO_2_ at 37 °C. For fluorescence imaging tests, we stained Carboxyfluorescein succinimidyl ester (CFSE) and Far Red from CellTrace™ for MCF-7 and NIH-3T3 cells. After the staining, MCF-7 and NIH-3T3 cells were washed with PBS, detached, centrifuged, and collected in each cell line in a 200 μL media solution with a total cell density of 3 × 10^7^ cells/mL.

### 2.4. Generation of Uniform-Sized 3D Tumor Spheroids

For the generation of spheroids, the cells in the growth medium were adjusted to the required cell concentration using a growth medium and injected into the microfluidic device using a syringe pump. To encapsulate cells into microdroplets, the fluorinated oil (Novec7500, 3 M, Maplewood, MN, USA) mixed with 2% diluted surfactant (Pico-Surf™, Dolomite Microfluidics, Royston, UK) was used. Encapsulated droplets containing cells were collected at the outlet of the microfluidic device and incubated for an additional 1 day in the cell incubation system (MCO-18 AC, Panasonic Healthcare Co., Ltd., Tokyo, Japan) for spheroid formation within the droplets. These spheroids were imaged under an optical microscope (IX73, Olympus, Japan) and the image was analyzed using Image J (National Institute of Health, Bethesda, MD, USA) software.

### 2.5. Spheroid Viability Assay

Incubated spheroids were transferred on to 96-well cell plate and cultured for 2 h in a medium containing Doxorubicin hydrochloride (Dox-HCl, Sigma-Aldrich, St. Louis, MO, USA) at concentrations of 2, 4, 6, 8, and 10 μg/mL. After Dox treatment, the spheroids were stained to measure viability using Live/Dead Cell Assay kit (Sigma-Aldrich, Bayswater, Australia) with staining for 20 min at 37 °C. The stained spheroids were imaged under the fluorescence microscope (IX73, Olympus, Japan) and the Live/Dead staining results were evaluated using Image J by the threshold function.

### 2.6. Reverse Transcription Polymerase Chain Reaction (RT-PCR) Assay

Homotypic and heterotypic MCTs were transferred into a petri dish and cultured for 1 and 5 days in vitro. The MCTs were gently retrieved and subsequently resuspended in a culture medium and then centrifuged at 1300 rpm for 3 min. Digestion in Trizol (Invitrogen, CA, USA) was performed, followed by chloroform extraction and precipitation with isopropyl alcohol. cDNA was synthesized from 2 micrograms of total RNA using PrimeScriptTM 1st strand cDNA synthesis kit (TAKARA, Shiga, Japan) according to the manufacturer’s instructions. One microliter of the cDNA reaction mixture was subjected to PCR amplification using gene-specific primers (Table 1), the AccuPower^®^ PCR PreMix (BIONEER, Daejeon, Korea). The PCR conditions were 95 °C for 5 min followed by above 25 cycles at 95 °C for 30 s, 56 °C for 30 s, and 72 °C for 30 s. PCR products were analyzed with a 1.5% agarose gel. Images of the RT-PCR ethidium bromide-stained agarose gels were acquired with an Olympus High-Performance CCD camera (Olympus Corporation, Shiga, Tokyo, Japan), and the quantification of the bands was performed with Image J. Mean and standard deviation of all experiments performed were calculated after normalization with GAPDH.

### 2.7. Statistical Analysis

Each experiment was performed at least three times. Data were expressed as means ± standard deviation. All statistical analysis was carried out using Prism version 8 (GraphPad Software, San Diego, CA, USA). A two-tailed unpaired t-test was performed comparing two groups, and one-way ANOVA with Tukey’s multiple comparison test for more than three groups. Differences between groups were considered statistically significant when *p* < 0.05 and are indicated with asterisks: * *p* < 0.05, ** *p* < 0.01, and *** *p* < 0.001.

## 3. Results and Discussion 

### 3.1. Design of the Microfluidic Gradient Droplet Device

The microfluidic-based droplet system described in this study allows robust yet simple 3D multicellular spheroid formation in a uniform-sized manner. This microfluidic droplet system is equipped with multiple inlets that facilitate the incorporation of varying cell types, thus allowing the control of the tumor microenvironment. In addition, the microfluidic-based droplet system consists of four tree-branched serpentine channels, thereby allowing a spontaneous cellular gradient to generate MCTs with various cellular ratios. The microfluidic platforms have previously been studied to regulate the long-term cell culture and precise handling of spheroids [16,27]. Although the previous microfluidic droplet devices were employed to generate the 3D spheroids, the throughput of the device was still very low and the system complexity was high [28,29]. In addition, their microfluidic designs were insufficient for precisely controlling in vivo tumor microenvironments. 

**Figure 1:**
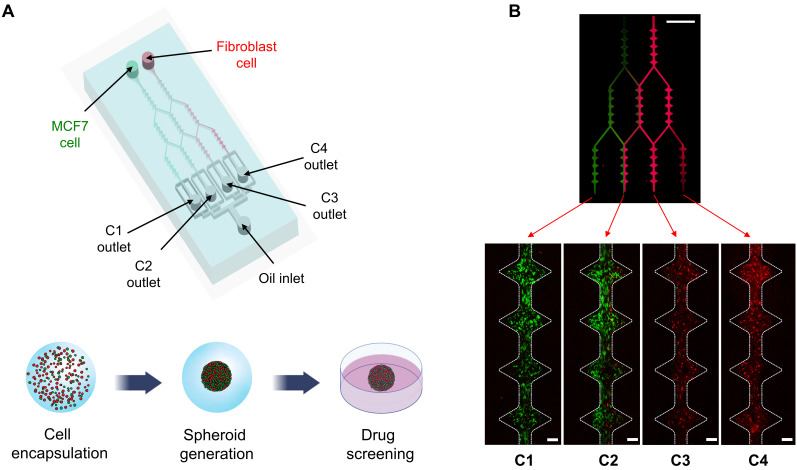
Experiment setup of microfluidic gradient droplet device for gradient-sized tumor spheroid generation. (**A**) Schematic illustration of gradient generation part and droplet generation part. Schematic 3D co-culture process of MCF-7 cells and fibroblasts. (**B**) Fluorescent image of gradient generation section.

Herein, we describe a microfluidic droplet approach allowing the generation of multicellular tumor spheroids with various stromal cell ratios in a single microfluidic system (Figure 1A). We observed the actual flow gradients in the microchannel; fluorescent molecules (FITC and Rhodamine) were used as a substitute to visualize the flow gradient generated in the microfluidic droplet chip. The developed fluorescent molecule gradients, after stabilizing for 30 min, were as shown in Figure 1B (upper panel). We also confirmed the actual cell gradient in microfluidic channels by labeling the MCF-7 with green fluorescent and fibroblast with red fluorescent using the CellTrace (Figure 1B, lower panel). We observed a lower diffusion flux than flow gradients because the fluorescently labeled cells were larger than the fluorescent molecules. Despite the cell gradient-developed region being narrower than the flow gradient, our system allows the massive generation of MCTs consisting of various cellular ratios. 

### 3.2. Computational Simulation and Cell-Encapsulated Droplet Generation 

**Figure 2:**
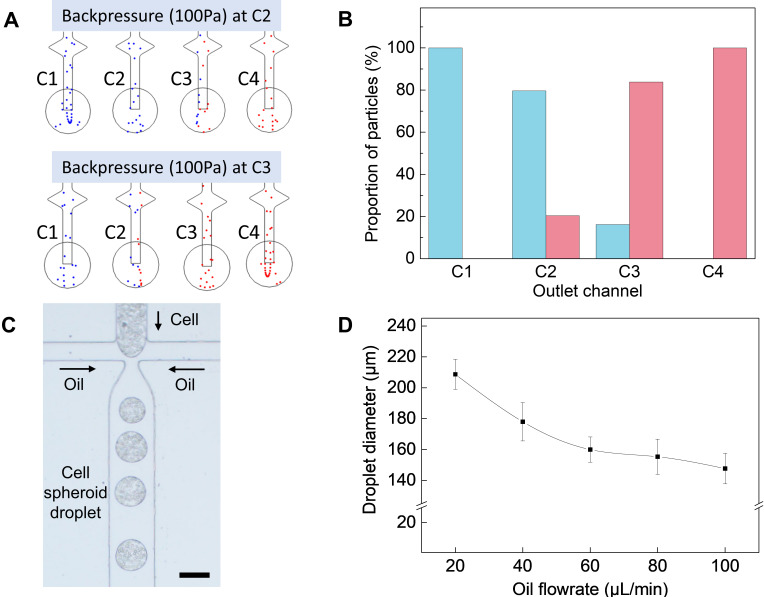
(**A**) COMSOL simulation of particle analysis for cell gradient. (**B**) Graph showing blue and red fraction of particles from each outlet. (**C**) Microscope image of encapsulated cell droplets generated using the microfluidic gradient droplet system. (**D**) Graph of cell droplet diameter with respect to oil flow rate at constant aqueous phase.

The mechanism of generating the fraction gradient was illustrated via computational simulation (Figure 2A,B). The associated physical concepts for this simulation involved: (1) the migration of the particles due to the drag force generated by laminar flow; (2) back pressure occurring during droplet generation; and (3) fraction gradients based on Y-shaped channels. First, the particle trajectories in a laminar flow were determined by drag force. As can be seen in Equation (2), the drag force is defined as a function of velocity unless the particle size changes. Thus, the particles in a laminar flow were significantly affected by velocity gradients and they migrated from the place with low velocity to the place with high velocity. It can be elucidated that the particles tend to migrate to where a more potent force is applied. Second, the droplet generation generally accompanies back pressure, which supplies the driving force in the opposite direction from the overall flow. Back pressure causes a drop in velocities and generates velocity gradients between the microchannels. We investigated the migration of particles according to where the back pressure occurred (Figure 2A). The blue and red particles were modeled as cell models of a cancer cell (MCF-7) and fibroblast. As aforementioned, since the velocity decreased in the microchannel with the back pressure, the drag force was relatively low. Hence, the particles migrated to the microchannel that didn’t experience back pressure. Third, assuming that the back pressure sporadically occurred between the microchannels and its frequency of occurrence was constant as previously reported [30], the particles were likely to migrate while generating their fraction gradients along the Y-shaped microchannels (Figure 2B). We counted the total amount of particles at each outlet for 5 min and calculated the fractions thereof. The fractions of blue particles were 100%, 83.8%, 20.4%, and 0%, respectively. 

To generate the multicellular spheroids encapsulated in droplets, cells suspended in a culture medium were used as the aqueous phase solution. In our device, droplet formation occurred at flow-focusing, where the aqueous inlet channel perpendicularly intersected the continuous oil phase channel (Figure 2C). We encapsulated cells in droplets under various flow rates. The droplet diameters were determined by the flow rates as shown in Figure 2D, and the diameter of droplets showed an inverse relationship with flow rates. In the initial phase, a non-uniform size of droplets formation was observed due to the mechanical limits of the experimental equipment, such as the unstable flow rate from the syringe pump. Thus, we optimized the flow rate of the aqueous and oil phase solution to 10 μ/min and 100 μL/min to generate the uniform size of droplets. By adjusting the fluid flow rates, the optimum droplets with an average size of 150 μm were obtained. We fabricated at least 2000 droplets per minute with the optimized flow rate set to generate the uniform size of droplets. 

### 3.3. Generation and Characterization of the Homotypic and Heterotypic MCTs

**Figure 3:**
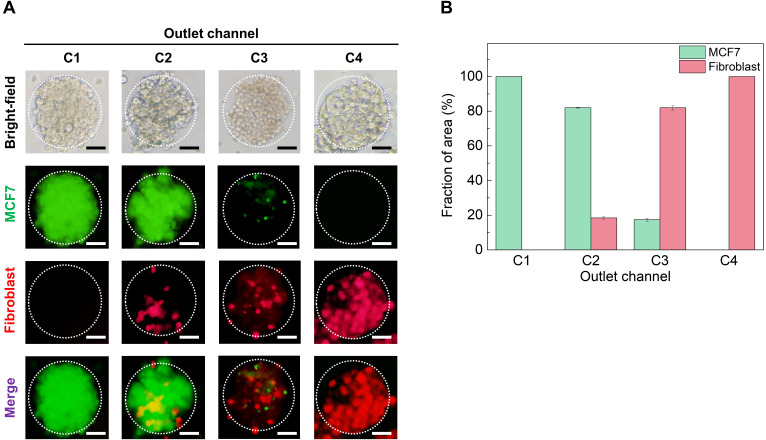
Multicellular spheroids within a droplet formed by the microfluidic gradient device. (**A**) Encapsulated cell images (MCF-7 and fibroblast cells were stained by CellTrace™ CFSE and CellTrace™ Far Red, respectively). Scale bars are 100 μm. (**B**) Graph of relative percentage of cells in encapsulated droplet by each outlet.

Herein, we used the MCF-7 and NIH-3T3 cell lines for the establishment of tumor spheroids. The MCF-7 cells are hormone-responsive to the breast cancer cell line [31], and NIH-3T3 cells were used in this study as a stromal cell, which can occupy a major portion of the tumor microenvironment [32]. We compared the computational simulation of particle proportion with the actual cell fraction area of homotypic and heterotypic MCTs. We labeled MCF-7 and fibroblast with different fluorescent molecules for the visualization of each cell. The MCF-7 cell line was stained using CFSE CellTrace and the fibroblast cell line (NIH-3T3) was labeled with a Far Red CellTrace to obtain a clear distinction between the two cell populations. Both CellTrace molecules readily diffuse into cells and bind covalently to intracellular amines in proteins resulting in stable and well-retained fluorescent staining [33]. A quantitative analysis measured the color intensity of the green and red fluorescence in Figure 3A, as shown in Figure 3B. The actual cellular fraction area of MCTs collected from each outlet corresponded with the computational simulation result. Therefore, our microfluidic-based droplet system was proven useful for the generation of homotypic and heterotypic MCTs which could form various ratios of fibroblast cells. 

### 3.4. Anti-Cancer Drug Screening Analysis

**Figure 4:**
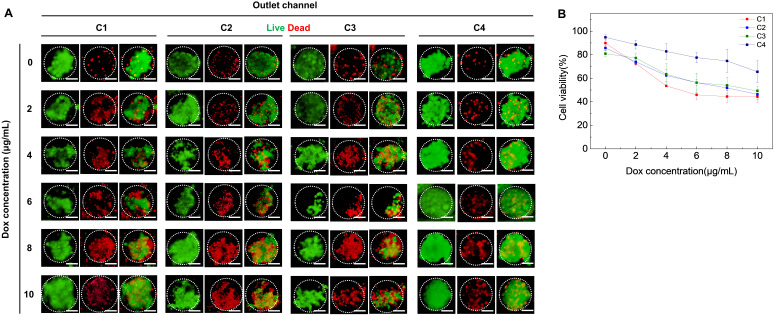
Spheroid generation after 1 day encapsulation. (**A**) Spheroid image after Dox treatment (live and dead cells were stained with Calcein-AM and EthD-1, respectively). Scale bars are 100 μm. (**B**) Cell viability with respect to Dox concentrations.

In our work, the MCF-7 and fibroblast cells were directly mixed and generated into multiple types of MCTs—homotypic and heterotypic—in the microfluidic-based gradient droplet system. To validate the generated MCTs for drug-based cytotoxicity screening, the standard chemotherapeutic drug (Dox) was utilized as a model agent. Dox acts by intercalating with the DNA of the cell to inhibit the action of the enzyme topoisomerase II required for supercoil relaxation during transcription [34]. Cytotoxicity results from the inhibition of replication and free radical formation. The cytotoxicity of Dox on multicellular spheroids were evaluated at various dosages, respectively, and the results are shown in Figure 4. The aim of our study is to compare and contrast the difference in cytotoxicity profiles in MCTs, which can contain different cell ratios. Fluorescent microscopic images of MCTs (Figure 4A), treated with an increasing concentration of Dox, were used for end-point cell viability validation using a Live/Dead assay. Two fluorescent dyes are employed to differentiate between live and dead cells, live cells represented by a bright green fluorescence and the nuclei of dead cells represented by a red fluorescence. The results indicated a dose-dependent decrease in cell viability as the cells were treated with increasing concentrations of Dox in MCTs (Figure 4B). Particularly, the cytotoxicity observed in MCTs containing fibroblast cells was markedly less as compared to the MCTs formed with MCF-7 cells only (Figure 4B). Furthermore, MCTs, which could consist only of MCF-7 cells treated with high concentrations of Dox (6 or 8 μg/mL) demonstrated a difference in cell viability between MCTs with fibroblast cells. This suggests that the fibroblast-free MCTs can be more susceptible to drug treatment. This result demonstrated that the presence of NIH-3T3 cells also enhanced the Dox resistance of MCTs at all drug concentrations (2–10 μg/mL). Thus, we confirmed that the cell viability was affected by the cellular ratio, showing that the more stromal cells contained, the more drug resistance increased. 

Drug resistance is developed and regulated by various causes in tumor microenvironments [35]. One important process is the contact between tumor cells and the stromal cells, which reconstitute the tumor structure and trigger multiple anti-apoptotic pathways in tumor cells through surface receptor-like integrins, which can significantly increase drug resistance [36,37]. In addition, stromal cells (e.g., fibroblast cells) play a key role in altering the anti-tumor activity of anti-cancer agents by cytokine and adhesion-mediated mechanisms [38]. However, conventional anti-cancer drug screening rarely employs other additional cell types during the evaluation of drug cytotoxicity. The efficient 3D model used to investigate drug resistance in cancer research consists of multicellular spheroids [39,40]. In addition, multicellular spheroid model complexity can be enhanced by incorporating multiple cell types (e.g., fibroblasts [41,42], endothelial cells [43], macrophages [44,45]). This structure mimics the increased cell–cell interaction and 3D transport gradient, similar to in vivo solid tumors [46]. Therefore, we demonstrated that the complexity of a 3D MCTs model (e.g., high density of cells, tight cell-to-cell contact) might act as the barrier to anti-cancer drug diffusion in vitro. Thus, our MCTs generated in the microfluidic-based droplet system monitoring the interaction of fibroblasts with tumor cells in a 3D microenvironment can allow us to optimize drug therapy protocols by assessing the contribution of these cells to cancer cell survival.

### 3.5. ECM Gene Analysis

**Figure 5:**
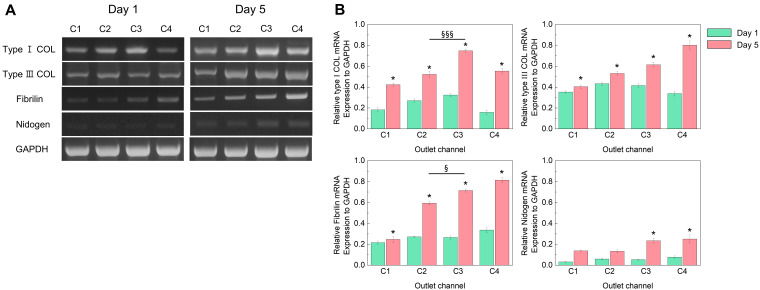
(**A**) Representative agarose gel showing mRNA expression of ECM-associated genes in MCTs generated from droplet system for days 1 and 5. (**B**) Quantification of expression of Type Ⅰ and Ⅲ collagen, fibrilin, and nidogen relative to GAPDH from day 1 to day 5. Data are represented as mean ± standard deviation of 3 independent experiments. * indicates *p* < 0.05 for day 1 vs. day 5, § indicates *p* < 0.05 for C2 vs. C3 in day 5, and §§§ indicates *p* < 0.001 for C2 vs. C3 in day 5.

We analyzed the cell–ECM interaction of homotypic and heterotypic MCTs by evaluating the expression of ECM gene-related markers from day 1 to 5 (Figure 5). Gene expressions in tumor homotypic and heterotypic MCTs were normalized with the housekeeping gene (GAPDH) and also compared with the MCTs on day 1. The significant upregulation of ECM-related genes on day 5 in comparison with day 1 was observed in homotypic and heterotypic MCTs (*p* < 0.05). Especially, all ECM-related genes showed the highest level in homotypic MCTs which consist of fibroblasts only, which might be quite relevant to the ratio of fibroblast cells. An interesting trend is observed for fibrilin and typeⅠcollagen gene modulation: in MCTs gathered from the C3 outlet, both genes showed significant upregulation as compared with C2 outlets on day 5 (up to 1.4 fold). Stromal fibroblasts are known to play key roles in the tumor microenvironment. Fibroblasts are activated at the site of tissue injury or inflammation, which presents a similar pathophysiological condition as seen in tumors. Increased fibroblast growth and activity have been observed in solid tumors [47]. In addition, the tumor in vivo is not merely an aggregation of cancer cells but a complex entity in which cancer cells interact with stromal cells and the ECM and together contribute to drug resistance [48,49,50]. The cellular components of tumor microenvironments interact with tumor cells and impact various biological characteristics, such as proliferation, migration, and therapeutic resistance [51]. Typically, the golden standard for in vitro tumor recapitulation is the spheroid, since it exhibits various biological properties of solid tumors including cell morphology, growth kinetics, and gene expression [52]. Spheroidal architecture is the most resembling tumor spatial organization in vivo, however, they cannot recapitulate the tumor microenvironment due to a lack of interaction with the ECM which has an important role in tumor progression and chemoresistance. Thus, it becomes important to incorporate the cellular components of tumor stroma in 3D models to obtain a more reliable drug screening tool [10]. 

Our results showed the overexpression of tumor microenvironment markers when the fibroblasts were co-cultured with MCF-7 cancer cells. The upregulation of ECM-related genes is an important phenomenon to confirm, as they play a critical role in the remodeling and progression of the tumor microenvironment. Under in vivo situations, the cancer cells in a solid tumor can acquire anti-cancer drug resistance through interaction with the ECM, such as collagen, laminin, and fibronectin [53]. Moreover, our heterotypic MCTs showed that cancer cells could stimulate the production of collagen by fibroblasts. The fibroblasts are a significant ECM source in normal and malignant tissue. However, the ECM in tumorous tissues differs notably from that in normal tissues and cancer cells produce a substantial amount of the ECM during the cancer progression, which has constituent ratios different from those of normal tissues. The ECM secreted by stromal cells also has a peculiar profile characterized by the overexpression of MMPs, collagen I, and fibronectin, among others. Those genes are hallmarks of the fibrotic phenotype in solid cancers [54,55]. In addition, laminin 5, hyaluronan, and TNC are highly expressed in cancer cells [56,57]. Changes in the content, composition, and organization of the ECM in tumorous tissues could contribute to drug resistance due to physical resistance to diffusional transport, and well-organized collagen fiber tends to make a stiff ECM, resulting in increased chemical protection [58,59]. A number of studies reported that ECM proteins could protect a variety of cancer cell lines from cytotoxic drug therapy in vitro [60,61,62]. Based on our results, MCTs containing stromal cells demonstrated ECM-mediated drug resistance and their contribution even at a microscale.

## 4. Conclusions

Our platform aims to be used as a tool for MCT generation with various cellular ratios for drug screening or investigation of the tumor microenvironment. Spontaneous gradient could easily and precisely control the cellular ratio of generated MCTs, which could facilitate the investigation of cell–cell and cell–ECM interactions. We generated four types of MCT models: homotypic MCTs (consisting of only MCF-7 or fibroblast cells), and heterotypic MCTs (consisting of 20% or 80% fibroblast per total percentage of the cellular fraction) to mimic the tumor microenvironment. To prove the interplay of cell–cell and cell–ECM, we conducted cytotoxicity experiments using a standard chemotherapeutic drug utilized in breast cancer treatment and compared the results to each MCT. The flexibility of the microfluidic-based droplet design can be further exploited to include various types of stromal components capable of remodeling the tumor microenvironment to investigate the drug effect. In addition, MCTs generated in this system may also be useful for a better understanding of chemoresistance mechanisms or metastatic spread.

## Figures and Tables

**Table 1:** Primer used for the analysis of expression by PCR.

Gene	Sense and Antisense
Type Ⅰ COL	5′-CCCCCTCCCCAGCCACAAAG-3′
	5′-TCTTGGTCGGTGGGTGACTCT-3′
Type Ⅲ COL	5′-TGGTCCTCAGGGTGTAAAGG-3′
	5′-GTCCAGCATCACCTTTTG GT-3′
Fibrilin	5′-ACGGCTTTACTGGACCCCA-3
	5′-ACATCTGGTTGCTTACCACAG-3
Nidogen	5′-AGGAGCTCTTTCCCTTCGGC-3′
	5′-CGGGGGTTCACTCGTAGCAA-3′
GAPDH	5′-TGACGTGCCGCCTGGAGAAA-3′
	5′-AGTGTAGCCCAAGATGCCCTTCAG-3′

## Data Availability

Not applicable.
